# Supplementary figures and images for: Genome-Wide Investigation and Functional Verification of the ZIP Family Transporters in Wild Emmer Wheat
Source: Int J Mol Sci. 2022 Mar 5;23(5):2866. doi: 10.3390/ijms23052866 (PMC8911026; doi:10.3390/ijms23052866)

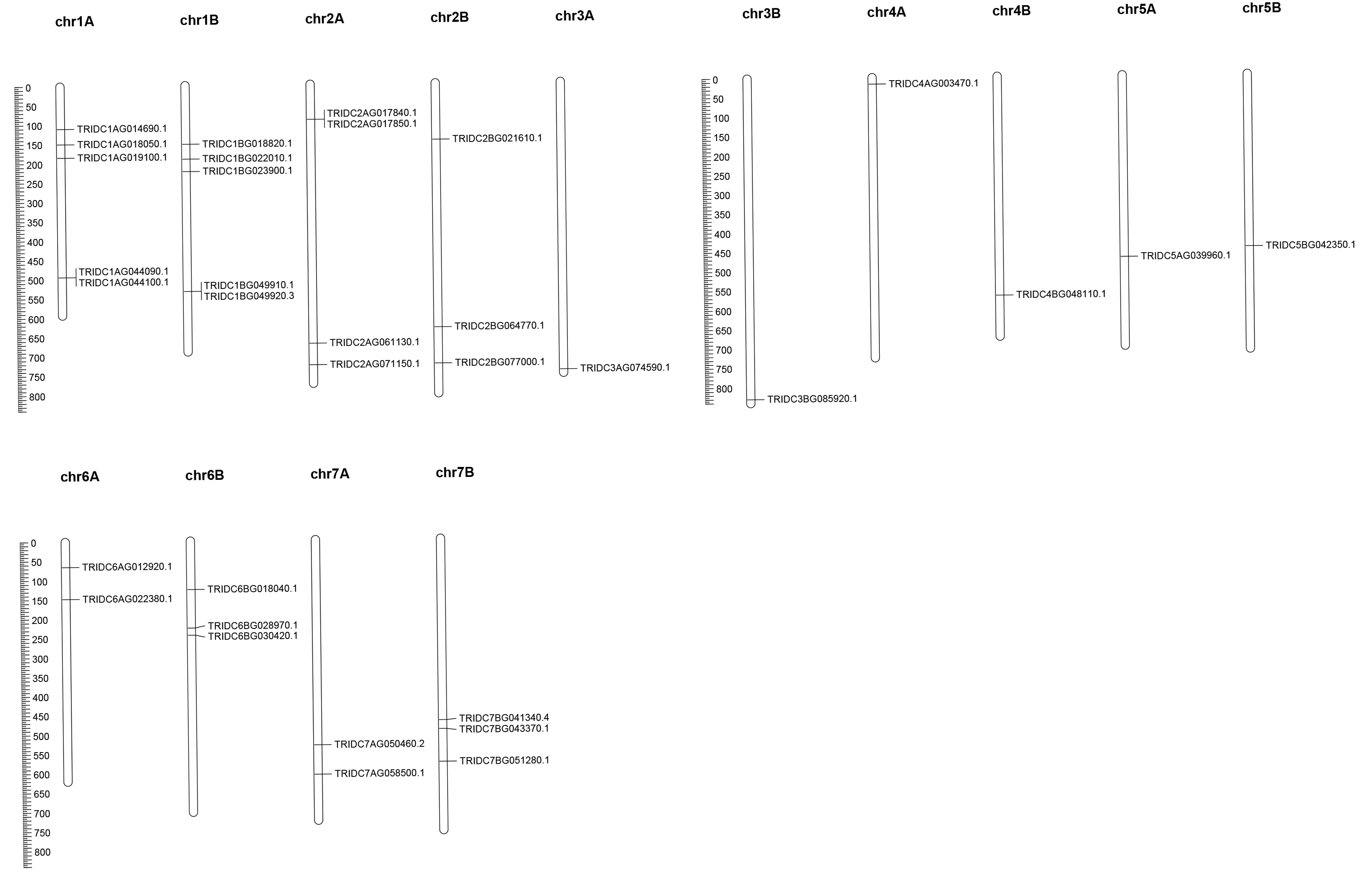

Supplement: Supplementary file 1 [file ijms-23-02866-s001.zip › Figure S1.tif]

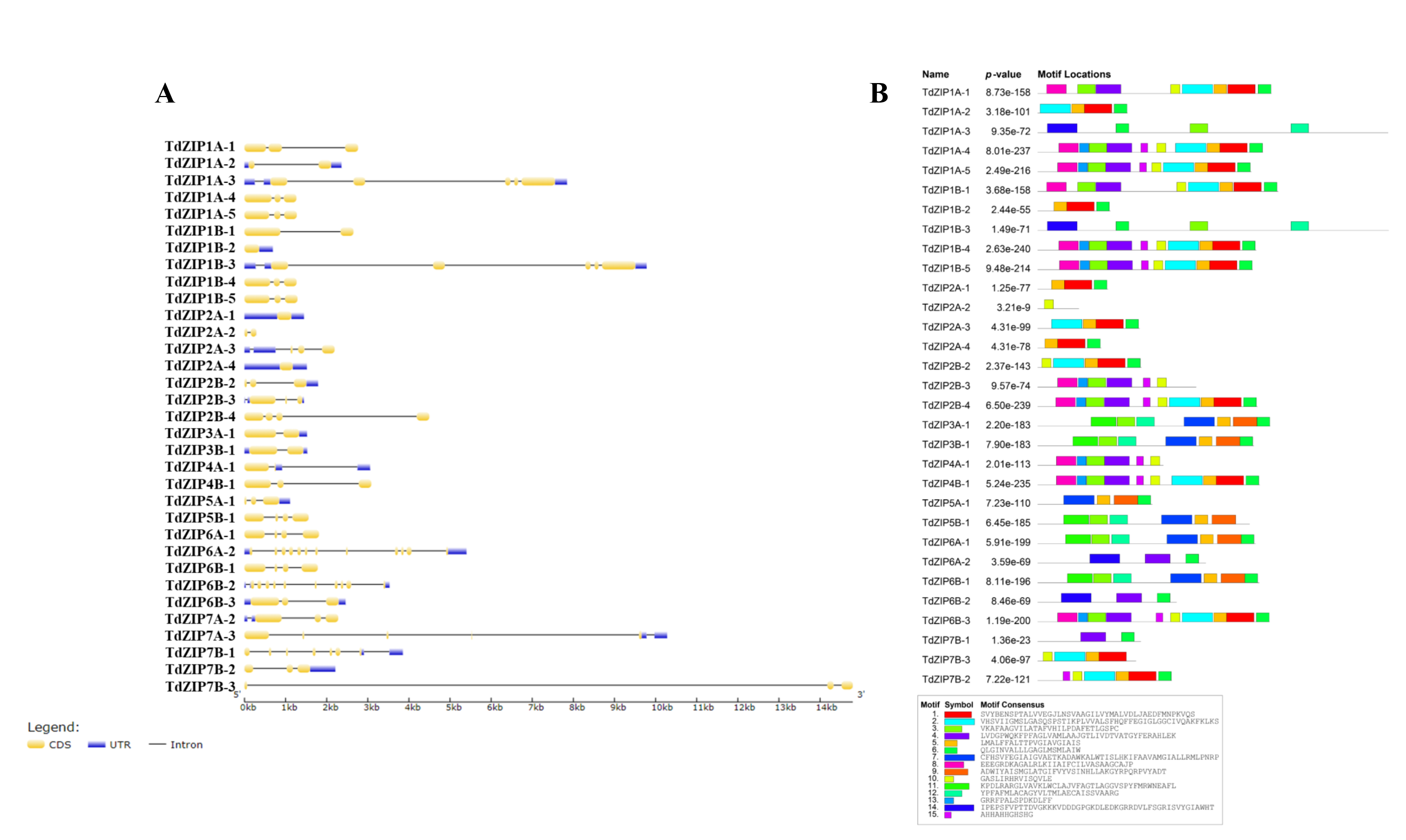

Supplement: Supplementary file 1 [file ijms-23-02866-s001.zip › Figure S2.tif]

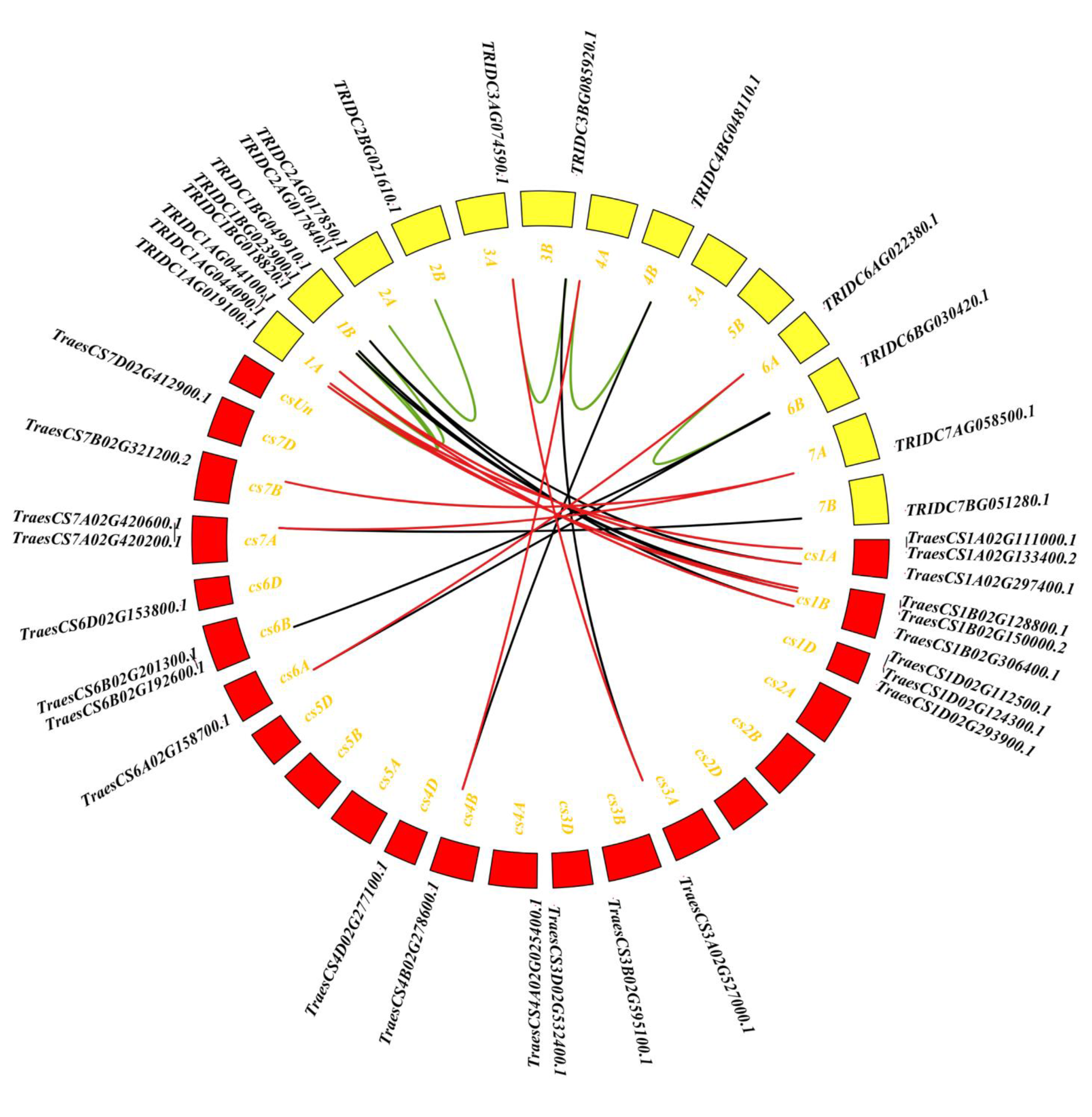

Supplement: Supplementary file 1 [file ijms-23-02866-s001.zip › Figure S3.tif]

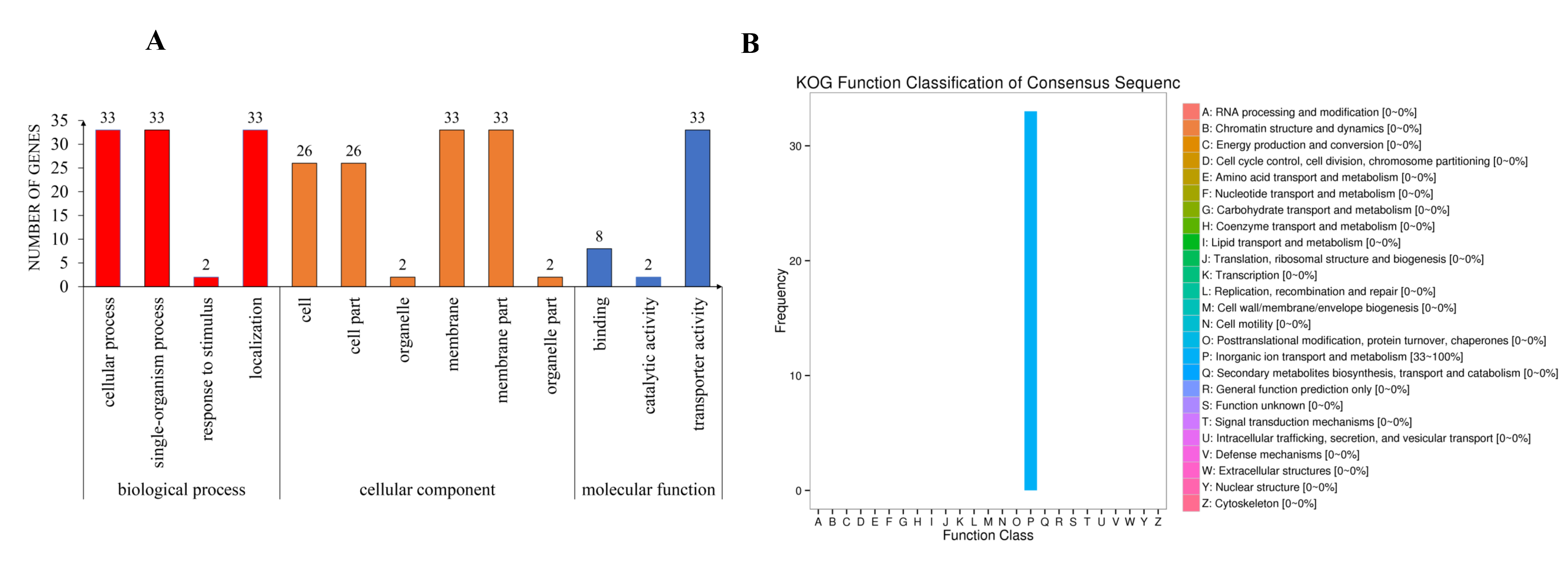

Supplement: Supplementary file 1 [file ijms-23-02866-s001.zip › Figure S4.tif]

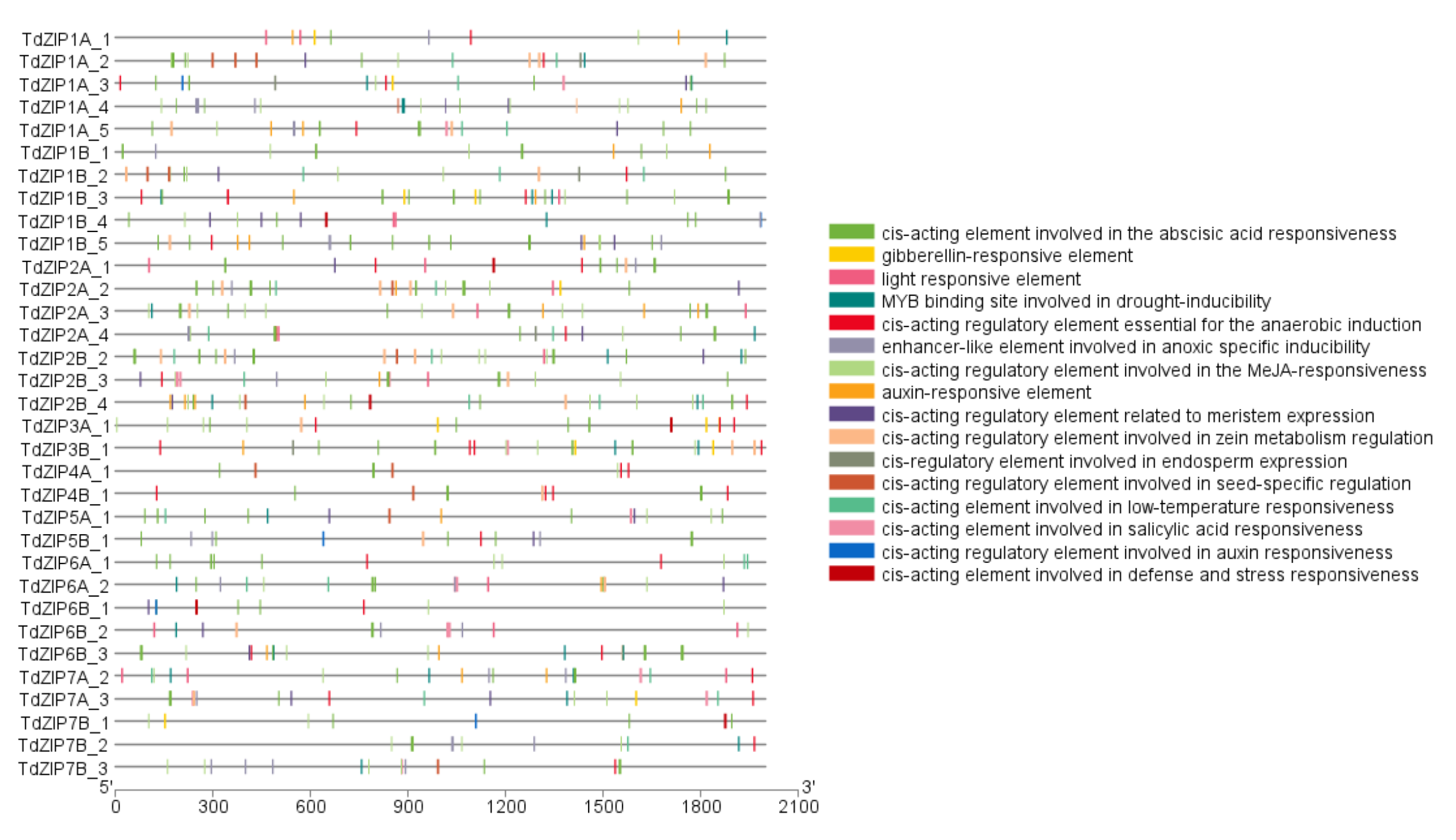

Supplement: Supplementary file 1 [file ijms-23-02866-s001.zip › Figure S5.tif]

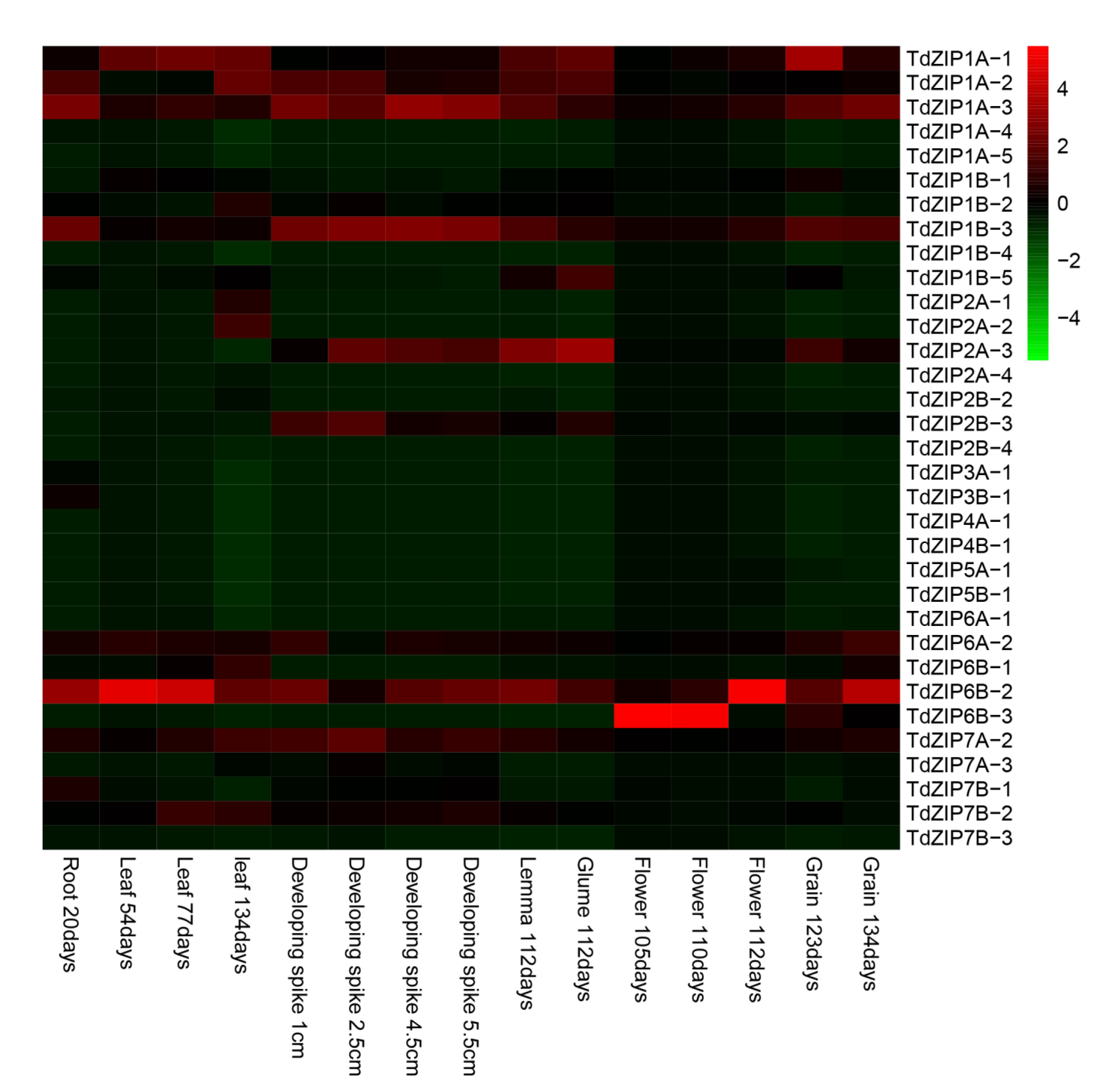

Supplement: Supplementary file 1 [file ijms-23-02866-s001.zip › Figure S6.tif]
